# Disruption-induced changes in syntrophic propionate and acetate oxidation: flocculation, cell proximity, and microbial activity

**DOI:** 10.1186/s13068-025-02644-3

**Published:** 2025-04-19

**Authors:** Nils Weng, Hossein Nadali Najafabadi, Maria Westerholm

**Affiliations:** 1 Department of Molecular Sciences, Swedish University of Agricultural Sciences, Uppsala, Sweden; 2 Department of Management and Engineering, Linköping University, 581 83 Linköping, Sweden

**Keywords:** Syntrophic propionate-oxidizing bacteria, Syntrophic acetate-oxidizing bacteria, Methanogens, Anaerobic digestion, Mixing, Flocculation, Computational fluid dynamics, Interspecies electron transfer

## Abstract

**Background:**

Syntrophic propionate- and acetate-oxidising bacteria (SPOB and SAOB) play a crucial role in biogas production, particularly under high ammonia conditions that are common in anaerobic degradation of protein-rich waste streams. These bacteria rely on close interactions with hydrogenotrophic methanogens to facilitate interspecies electron transfer and maintain thermodynamic feasibility. However, the impact of mixing-induced disruption of these essential syntrophic interactions in biogas systems remains largely unexplored. This study investigates how magnetic stirring and orbital shaking influence degradation dynamics, microbial community composition, and gene expression in syntrophic enrichment communities under high-ammonia conditions.

**Results:**

Stirring significantly delayed the initiation of propionate degradation in one culture and completely inhibited it in the other two parallel cultures, whereas acetate degradation was less affected. Computational fluid dynamics modelling revealed that stirring generated higher shear rates (~ 20 s^−1^) and uniform cell distribution, while shaking led to lower shear rates and cell accumulation at the bottom of the culture bottle. Visual observations confirmed that stirring inhibited floc formation, while shaking promoted larger flocs compared to the static control condition, which formed smaller flocs and a sheet-like biofilm. Microbial community analysis identified substrate type and degradation progress as primary drivers of community structure, with motion displaying minimal influence. However, metatranscriptomic analysis revealed that motion-induced gene downregulation was associated with motility, surface sensing, and biofilm formation in SAOB and another bacterial species expressing genes for the glycine synthase reductase pathway. Stirring also suppressed oxalate–formate antiporter expression in SPOB, suggesting its dependence on spatial proximity for this energy-conserving mechanism. The strongest gene expression changes of stirring were observed in methanogens, indicating a coupling of the first and last steps of hydrogenotrophic methanogenesis, likely an adaptive strategy for efficient energy conservation. Other downregulated genes included ferrous iron transporters and electron transfer-associated enzymes.

**Conclusions:**

This study highlights that stirring critically disrupts the initial syntrophic connection between SPOB and methanogens, whereas SAOB communities exhibit greater tolerance to shear stress and disruptive conditions that inhibits aggregate formation. These findings emphasize the importance of carefully managing mixing regimes, especially when attempting to reactivate ammonia-tolerant syntrophic propionate degraders in biogas systems experiencing rapid propionate accumulation under high-ammonia conditions.

**Graphical abstract:**

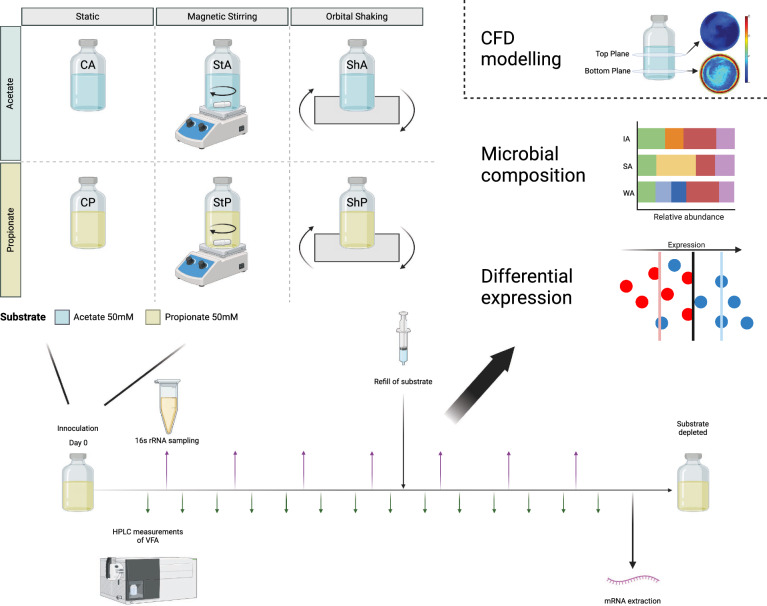

**Supplementary Information:**

The online version contains supplementary material available at 10.1186/s13068-025-02644-3.

## Introduction

To function effectively, biotechnological systems rely on a synergistic interplay between microbiology, chemistry, and technology. An example of this is the anaerobic digestion (AD) process, wherein productivity is directly linked to microbial activity which is, in turn, governed by both chemical conditions and process technology [1]. AD is a well-established biotechnology that converts organic waste into renewable energy (biogas), green chemicals, and sustainable fertiliser [2]. These products are vital components in the transition to a more carbon–neutral society, as they offer sustainable alternatives to fossil-derived fuels and chemicals. In addition, the use of sustainable fertilisers promotes nutrient recycling and reduces the reliance on mineral fertiliser, which is responsible for a significant amount of greenhouse gas emissions during both production and application [3–5].

From a microbial perspective, the AD process involves a continuous interaction between distinct microbial species which are responsible for transforming complex compounds into methane. This process is generally divided into four stages. In the first hydrolytic stage, proteins, fats, and carbohydrates are broken down into amino acids, fatty acids, and sugars. This is followed by the acidogenesis and acetogenesis stages, where these intermediates are further converted into volatile fatty acids (VFA), alcohols, ammonia, carbon dioxide (CO_2_), and hydrogen (H_2_). During the last step, methane is formed from either acetate (acetoclastic methanogenesis) or by the reduction of CO_2_, typically using H_2_ or formate as a reducing agent (hydrogenotrophic methanogenesis). By taking chemistry and process technology into consideration, the microbial interplay becomes more complex, as the various species can be distinctly affected by certain process conditions. This is particularly evident during ammonia inhibition, which frequently occurs in AD processes that degrade protein-rich materials, such as chicken manure or slaughterhouse waste. Although the use of protein-rich substrates offers a high methane yield potential and produces ammonia-rich digestate with a significant value as a fertiliser, elevated ammonia levels can inhibit key members of the microbial community. Acetoclastic methanogens, which convert acetate to methane, are notably sensitive to elevated ammonia concentrations [6–8], and their reduced activity under these conditions leads to an acetate build-up. Moreover, the inhibition of microorganisms that are responsible for degrading other acids, such as propionate, often results in additional VFA accumulation [9]. This further diminishes process performance and methane yield and can, in severe cases, lead to a complete process failure.

At high ammonia levels, an alternative route for acetate conversion to methane frequently emerges, where syntrophic acetate oxidising bacteria (SAOB) oxidise acetate into CO_2_ and H_2_. For propionate degradation, syntrophic propionate oxidising bacteria (SPOB) convert propionate into acetate, CO_2,_ and H_2_. The formed acetate can then be used by either acetoclastic methanogens in low ammonia conditions [10, 11] or by SAOB under high ammonia conditions [12, 13]. While SAOB are crucial for the AD process under high ammonia conditions, SPOB convert propionate in both low and high ammonia conditions, although the genera of SPOB observed at high ammonia levels typically differ from those that are active under lower ammonia conditions [12, 14].

Both the acetate and propionate oxidation reactions are exothermic under standard conditions and rely on a hydrogenotrophic methanogenic partner to consume the products, thereby making the reaction thermodynamically feasible. This mutualistic cooperation involves the acid oxidiser producing an excess of reducing equivalents during oxidation, which the methanogen uses to reduce CO_2_. These reducing equivalents are subsequently transferred through a mediated interspecies electron transfer using either H_2_ or formate [15, 16]. Direct electron transfer between cells has also been suggested as a possible mechanism; however, the extent to which this occurs during syntrophic acid oxidation is currently unclear. For mediated transfer, formate is considered to be more efficient than H_2_ for electron transfer over longer intracellular distances, due to its higher solubility but lower diffusivity [15, 17]. However, regardless of whether H_2_ or formate is used for electron transfer, close proximity between the syntrophic bacteria and the methanogen enhances the efficiency of transferring reducing equivalents between the cells.

The formation of flocs, also referred to as flocculation, is a common microbial strategy to reduce cell-to-cell distance. This has been observed in mesophilic syntrophic acetate and propionate oxidising enrichment cultures under high ammonia conditions [13], as well as in other syntrophic cultures cultivated under mesophilic and low ammonia conditions [18]. The importance of cell proximity was also highlighted in a study of high ammonia mesophilic enrichment cultures, which found a higher abundance of syntrophs within flocs compared to platonic cells [19]. Furthermore, in low ammonia and thermophilic conditions, microscopic observations revealed that a SPOB and a hydrogenotrophic methanogen grew as free-living cells in monoculture but co-aggregated when cultivated together in a syntrophic propionate degrading coculture [20]. These results raise the question of whether the observed flocculation is primarily driven by its role in syntrophic acid degradation or if it arises from other reasons commonly associated with microbial aggregation, such as protection from environmental stress, nutrient cross-feeding, exchange of genetic material, or attachment to solid surfaces [21–24]. The protective function is particularly relevant in high-ammonia environments, where microbial communities must withstand significant stress. This underscores the importance of integrating microbiological insights with process technology, as reactor mixing can significantly influence microbial flocculation.

In AD, rapid mixing has been suggested to disrupt spatial proximity between hydrogen producers and consumers [25], and it has also been proposed to negatively impact propionate degradation [26]. Although the effects of mixing on the biogas community and process performance [27, 28] has been previously investigated, no studies have specifically examined the impact on syntrophic acid oxidising communities, which are highly reliant on close cell proximity for optimal function. Studying syntrophic interactions within the complex web of microbial interactions of the AD process makes it challenging to isolate the effects of mixing on syntrophic acid oxidation, from those on other stages in the process, such as acid production rates. In addition, the low abundances of the syntrophic community commonly occurring in AD [29] obstructs studying the effects on a molecular level.

The objective of this study is to investigate how disruptive motion affects the activity of syntrophic acid-oxidizing communities under high-ammonia conditions. Specifically, we aimed to determine whether harsh stirring disrupts flocculation, impact on the acid degrading activities, and alters electron transfer mechanisms. We hypothesized that obstructed flocculation would lead to a lower expression of biofilm-associated genes and a shift in electron transfer mechanisms toward formate-mediated transfer. To test this, we used mesophilic enrichment cultures degrading either acetate or propionate under high-ammonia conditions and subjected them to two different mixing modes: magnetic stirring and orbital shaking. The impact of these conditions on degradation rates, microbial community composition, and microbial metabolic activities were analysed using molecular analyses. Furthermore, computational fluid dynamics (CFD) modelling was employed to assess the hydrodynamic forces and fluid motion generated by different mixing modes. CFD modelling provided insights into variations in shear rates and particle distribution, serving as a proxy for microbial dispersion. Given the essential role of syntrophic communities in preventing VFA accumulation in high-ammonia biogas reactors, understanding their response to mixing can help optimize VFA degradation and improve biogas process stability.

## Materials and methods

### Source of microbial community and batch cultivation setup

The syntrophic propionate-oxidising and acetate-oxidising enrichment cultures were derived from mesophilic laboratory-scale continuous stirred-tank reactors (CSTRs), previously described by Singh et al. [12]. In short, these CSTRs were inoculated with sludge from a high-ammonia biogas reactor degrading food waste and supplemented with albumin. The CSTRs were continuously fed with a bicarbonate-buffered medium containing ammonium chloride (0.3 M) and sodium propionate (0.1 M) as substrate [9]. The CSTRs were operated for over 144 days before the enrichment cultures were transferred to anaerobic serum bottles.

The anoxic bicarbonate-buffered medium was prepared as described by Westerholm et al. [30] and supplemented with ammonium chloride (0.3 M NH_4_Cl). However, unlike previous publications [12, 13, 19] the enrichment cultures in this study were cultivated through multiple sequential transfers into medium without the addition of yeast extract. This repeated transfer process ensured the gradual elimination of all carbon sources except for the acids (excluding the reducing agent cysteine).

For the preparation of cultivation batches for this study, 0.225 L of the medium without yeast extract containing either 50 mM sodium acetate (**A**) (C**A**, St**A**, Sh**A**) or 50mM sodium propionate (**P**) (C**P**, St**P**, Sh**P**) was transferred to serum bottles (0.5 L) while flushing with N_2_. At this stage, stirrer magnets were added to the bottles designated for stirring motion (**St**) (**St**P, **St**A). The bottles were each sealed with a butyl stopper and the gas phase was exchanged to N_2_/CO_2_. The bottles were autoclaved at 121 °C for 20 min, then allowed to cool to room temperature, before adding sterile-filtered solutions containing vitamins, trace elements, and the reducing agents cysteine–HCL (0.5 g/L final concentration) and Na_2_S (0.24 g/L final concentration), reaching a working volume of 0.25 L. The batches were inoculated with 12.5 mL (5% v/v) of the aforementioned enrichment culture grown in a medium without yeast extract. Triplicate batches were incubated for each setting in the dark at 37 °C. The control batches (C) were incubated under static conditions. Batches designated for shaking (**Sh**) (**Sh**A, **Sh**P) were placed on an orbital shaker (Orbitron Orbital Schüttler Shaker, Infors HT, Switzerland) with a circular motion radius of 14.5 mm and a frequency of 2Hz (120 rpm). Batches designated for stirring (StA, StP) were placed on magnetic stirrers set to an approximate speed of 700 rpm. The stirring speed was estimated by analysing slow-motion video footage of the magnetic stirrer, counting revolutions, and dividing by the video recording time. The final rotational speed was calculated as the average of three separate recordings. Consequently, in this setup triplicate batches were exposed to either: static (C), gentle shaking under relative mild conditions (Sh) and intense, high-speed stirring conditions (St). Once the initially supplemented substrate was depleted, it was replenished to a concentration of 50 mM on day 111 for acetate-fed cultures, and day 300 for propionate-fed cultures and again incubated under respective conditions.

### Chemical analytical methods

For chemical analysis, liquid (2 mL) and gas (1 mL) samples were extracted using syringes. Propionate and acetate levels were measured using high-performance liquid chromatography (HPLC), as previously described by Westerholm et al. [30]. Methane and CO_2_ composition of the headspace were measured using gas chromatography as described by Westerholm et al. [31]. Pressure measurements of the headspace were taken each time liquid and gas samples were extracted, using a handheld pressure meter (GMH 3111, Gresinger). Pressure measurements were also regularly taken to monitor whether acid degradation had started during the long lag phases of the batch cultivations.

### Optical inspection of floc-formation

Images were taken of the batch assays 56 and 104 days after incubation. Bottles were placed on a sheet of acrylic glass, and images were taken from beneath and from the side of the bottle.

To microscopically visualise aggregates and methanogenic activity (based on F_420_ autofluorescence) a droplet of sample culture was placed on a microscope slide. Micrographs were captured using a fluorescent microscope (Lumascope LS720, Etaluma) at 60 × magnification. F_420_ autofluorescence of methanogens was visualised using a 370–410 nm excitation filter and a 429–462 nm emission filter. Microscopic inspection was performed on all stirred propionate samples, and on a representative replicate from the control and shaking batch assays.

### Computational fluid dynamics modelling

A CFD model was constructed to determine the impact of stirring and orbital shaking on the hydrodynamic conditions and cell distribution. Two complementary simulations were constructed for each mode of motion. Light particles (1 µm) were used as a proxy to represent the cells and small-scale aggregates, which were initially homogeneously dispersed throughout the fluid. The cultivation batches in serum bottles were modelled up to the top of the working liquid height using finite element-based solver COMSOL Multiphysics (v 6.2) [32]. The stirring was modelled through a moving mesh with a domain rotation corresponding to 700 rpm. For the shaking motion, the same control volume used for stirring was applied, but without the magnet. Modelling was conducted in COMSOL in Multiphysics using mesh deformation with the following relations:

$$\begin{array}{lll} {\text{Mesh displacement in }}X &\qquad R\cos (2\pi \omega ) \hfill \\ {\text{Mesh displacement in }}Y & \qquad 0 \hfill \\ {\text{Mesh displacement in }}Z &\qquad R\sin (2\pi \omega ) \hfill \\ \end{array}$$

where *R* is the radius of rotation and ω the frequency with values of 0.0145 [m] and 2 [Hz], respectively.

The governing equations, conservation of momentum and mass, take the form of Reynolds Averaged Navier–Stokes (RANS) equations for turbulent flow. The Realizable $k-\varepsilon$ model has been used as the turbulence model, which considers two additional transport equations, namely the turbulent kinetic energy and dissipation to be solved along with RANS. This is the most commonly used turbulence model within the community to simulate the hydrodynamic environment in bioreactors [33, 34] and its performance was validated using Particle Image Velocimetry by Sucosky et al. [35]. The calculated Re are 6992 and 1597 for the stirring and orbital shaking table, respectively, which suggests turbulent flow as they are above the critical Re of 1000 [36]. The characteristic lengths used in the model were the length of the magnet stirrer and the translational motion diameter, defined as 2*R* for the orbital shaking-table. The cultivation medium, being an aqueous solution, was treated as water for the CFD analysis, and it was assumed that its density and viscosity were equal to water at 20 °C. A no-slip boundary condition was used for the walls, and in the case of the orbital shaker, the walls followed the frame motion. A symmetry plane was applied at the free surface of the liquid, and gravitational forces were accounted for as volume forces.

To investigate the effect of hydrodynamic forces on floc distribution, a phase transport module was employed to solve for the mass transport of two species; water as liquid and microbes or microbial aggregates as solid particles, with a diameter of 1 µm and 1% heavier than water, purposefully selected to approximate the size and density of microbial cells [13, 37]. The initial volume fraction of heavier particles was set at 0.1, with a homogenous distribution. The Multiphysics coupling between turbulent flow and phase transport was carried out through the Mixture Model from COMSOL Multiphysics. The selected slip and mixture viscosity models are Hadamard-Rybczynski and Krieger, respectively [32].

The computational mesh uses tetrahedral elements for the bulk flow and 4 layers of prism elements to capture the larger gradients near the wall. The grid convergence index [38], based on the volume average of shear rate and velocity, showed a GCI value of less than 1% for a mesh with the control volume consisting of 166,729 elements compared to a finer mesh. The selected mesh had element size distribution ranges from 4.63 × 10^–4^ to 9.83 × 10^–3^. The implicit time-dependent solver was run until the average volume values of velocity and shear rate changed by less than 1% for the last two consecutive times, indicating that the solution had reached a steady state. This was established after 20 s for the magnetic stirring and 50 s for the orbital shaker. For example, in the earlier case, the comparison of volume average shear rate and velocity between times 20 s and 21 s had indicated errors of 0.03% and 0.4%, respectively. The post-processing considered the hydrodynamic force, i.e., contours of shear rate and volume fraction of solid particles, denoting the distribution of microbes or microbial aggregates, which were initially uniform. Two circumferential planes located at the bottom and top of the computational domains were selected to study the effect of stirring or shaking.

### 16S rRNA gene amplicon sequencing, data analysis and qPCR

16S rRNA gene amplicon sequencing was conducted to monitor the microbial community structure over time. Due to differences in degradation dynamics between experimental setups, liquid samples (5 mL) were taken at various timepoints to obtain samples representing different stages of degradation. The samples were taken as follows: acetate degrading batch cultures (CA, ShA, StA) on days 49, 69, 97, 111, 125, and 139; propionate degrading control and shaking motion batch cultures (CP, ShP) on days 111, 139, 160, 216, 230, 251, 300, 328, and 338; stirred propionate culture (StP1) on days 196, 216, 230, 251, 280, 300, 328, and 338. Due to the absence of propionate degrading activity, the stirred propionate cultures StP2 and StP3 were sampled only on days 196, 251, and 300. The samples were stored at – 20 °C until DNA extraction. After thawing, total DNA was extracted using the DNAeasy Blood and Tissue Kit (Qiagen) according to the manufacturer’s instructions. Construction of 16S rRNA gene amplicon libraries using primers 515F [39] and 805R [40] and Illumina sequencing was performed as described by Muller et al. [41]. Paired-end sequencing was conducted using an Illumina MiSeq instrument (Eurofins, Germany) at SciLifeLab Stockholm, Sweden.

The raw sequencing data underwent a multistep pre-processing pipeline. Due to the initial poor quality of the reverse reads, the first 42 bases were removed from the reverse reads using Trimmomatic (v 0.39) [42]. Subsequently, primer and adapter sequences were removed using Cutadapt (v 4.7) [43]. Amplicon sequence variants and abundance tables were generated in R (v 4.2.1) using the *dada2* package (v 1.24.0) [44]. In the *dada2* pipeline, forward and reverse sequences were cut to 272 and 182 bp, respectively, with a quality threshold of maxEE = (2, 7) and a default value of truncQ = 2. For taxonomic assignment of the amplicon sequence variants, a reference database formatted specifically for *dada2* was utilised [45]. This database was derived from the Genome Taxonomy Database (GTDB) release 207 [46]. The *phyloseq* package (v 1.40) [47] was used for subsequent data handling and visualisation of the microbial community structure. Non-metric Multidimensional Scaling (NMDS) was applied to assess the similarity of the community structure between experimental setups. The distance matrix used for the NMDS was created using Bray–Curtis distance.

To compensate for the restricted specificity of archaeal 16S rRNA genes by the primers used in the present study [48], quantitative PCR (qPCR) analyses were conducted on samples taken from the propionate and acetate enrichment batches at the following timepoints: CA, ShA, and StA on days 49, 69, 126, and 139; CP and ShP on days 139, 196, 251, and 328; and StP1 on days 216, 251, 300, and 328. The qPCR was conducted using primers MMBf (5ʹ-ATCGRTACGGGTTGTGGG-3ʹ) and MMBr (5ʹ-CACCTAACGCRCATHGTTTAC-3ʹ) to determine the 16S rRNA gene level of methanogens of the order *Methanomicrobiales* [49]. Total bacteria were assayed using the forward primer (5ʹ-GTGITGCAIGGIIGTCGTCA-3ʹ) and reverse primer (5ʹ-ACGTCITCCICICCTTCCTC-3ʹ) based on primers described in Maeda et al. [50]. The qPCRs were performed in a 20 μL reaction mixture that consisted of 3 μL DNA sample, 10 μL ORA™ SEE qPCR Green ROX Master Mix (HighQu), 1 μL of each primer (10 μM). The qPCR protocol for quantification was as follows: 7 min at 95 °C, 40 or 55 cycles of 95 °C for 40 s, annealing at 66 or 61 °C (for the order *Methanomicrobiales* and total bacteria, respectively) for 1 min and 72 °C for 40 s, and melting curve analysis at 95 °C for 15 s, followed by 1 min at 55 °C and finally at 95 °C for 1 s. All reactions were carried out in a CFX Duet Real-Time PCR System (BioRad).

### RNA extraction, sequencing

For RNA extraction, the microbial cultures were sampled during the exponential phase of acid degradation, after the second substrate addition, to ensure sufficient biomass and active acid degradation. Sampling occurred on day 125 for acetate-fed cultures (CA, StA, ShA) and on day 338 for propionate-fed cultures (CP, StP, ShP). Total RNA was extracted using a chloroform/phenol-based method, followed by rRNA depletion using riboPOOL™ (as outlined in the RNA extraction and depletion protocol by Perman/Weng [51]). To sample any flocs formed during cultivation, bottles were gently inverted to allow floc sedimentation. For acetate-fed cultures, a single 50 mL sample was taken from each bottle and transferred to N_2_-flushed Falcon tubes that were kept on ice. For propionate-fed cultures, triplicate 50 mL samples were collected from each bottle and similarly transferred to N_2_-flushed Falcon tubes on ice. All tubes were centrifuged at 5000*g* and 4 °C for 10 min, the supernatant was discarded, and the cell pellets were pooled in 1 mL chilled TRIzol (Thermo Fisher Scientific, MA, USA) and 0.2 mL chloroform. For acetate-fed cultures, pooling was carried out on a biological replicate level, yielding one sample per motion type (CA, StA, ShA). For propionate-fed cultures, technical replicates were pooled, producing one sample per biological replicate (CP1–3, StP, ShP1–3). Two of the stirred propionate cultures did not degrade propionate (StP2, StP3) and failed to yield sufficient RNA for analysis. Resultingly, two technical replicates were extracted from the stirred propionate culture that did degrade the acid, herby referred to as StP1 and StP12. RNA extraction was performed using the Quick-RNA Fecal/Soil Microbe Microprep Kit (Zymo Research, CA, USA) with an additional Dnase I treatment step. Extracted RNA samples were stored at − 80 °C. Ribosomal rRNA was depleted using pan-prokaryote riboPOOL probes with streptavidin-coated Dynabeads (MyOne Streptavidin C1, Invitrogen #65001). Depleted RNA was purified using ethanol precipitation and stored at − 80 °C until submission for sequencing. RNA concentration and quality was assessed using a 2100 Bioanalyzer System with RNA 6000 Nano Kit (Agilent Technologies Inc., CA, USA). The rRNA-depleted RNA was sequenced using paired-end sequencing (2 × 150 bp) on an Illumina NovaSeq X Plus (Eurofins, Germany), using one lane of a 10B flow cell. Sequencing was conducted at the SNP&SEQ platform (SciLifeLab, Uppsala, Sweden).

### RNA analysis

Raw sequences were trimmed off adapters using Cutadapt (v 4.0) [43] and quality filtered using Trimmomatic (v 0.39-2) [42]. In silico-removal of rRNA was carried out using SortMeRNA (v 2.1b) [52]. Quality controlled and trimmed reads were then quantified through a reference-based approach using Salmon (v 1.9.0) [53]. Metagenome-assembled genomes (MAGs) from a previous study investigating the same enrichment culture were used for read mapping [13], and complemented with a single MAG affiliated to the genera *Alkaliphilus* extracted from the high ammonia CSTRs from which the present enrichment culture originates [12]. Quantification results were outputted as raw counts. Differential gene expression analysis was conducted in R using DESeq2 (v 1.36.0) [54] and results were visualised using the packages pheatmap (v 1.0.12) [55] and ggplot2 (v 3.5.0) [56]. For the main differential gene expression analysis investigating the effects of motion, only propionate fed samples were used. Genes with low expression (less than 2 samples with a gene count of at least 10) were sorted out prior to analysis. *P* values were adjusted (p-adj) using the Benjamin–Hochberg method [57]. Genes were assigned as differentially expressed if they had an absolute value of log twofold change (log2FC) > 1.5 and an adjusted *p* value of < 0.05. For the principal component analysis (PCA), genes with low expression were filtered out as described above and transformed using regularised-logarithm transformation (rlog) of the Deseq2 package.

GapMind [58] was used to identify likely biosynthetic pathways for all amino acids, the associated coding sequences (candidate genes) to each pathway, and the completeness of each pathway for each respective MAG. All reported candidate genes were included in the gene sets used for subsequent pathway enrichment analysis and heatmap visualisation. When multiple candidates were associated with a single gene, the aggregated count was displayed in the heatmaps. Pathway enrichment was tested assuming a hypergeometric distribution with the *p* value calculated as the probability of observing at least n pathway-related genes among the differentially expressed genes and was tested for up- and down-regulated genes separately. This was done using the R-basic stat function phyper (q−1, m, n, k, lower.tail = FALSE):

q = number of differentially expressed genes in pathway list.

m = number of genes in pathway list.

n = number of background genes not in pathway list.

k = number of differentially expressed genes.

where the background gene set was defined as all genes that passed the initial filtering for low expression (at least two samples with counts > 10). The *p* value was adjusted for multiple testing according to the Benjamin–Hochberg procedure [57].

## Results and discussion

### Degradation of acetate and propionate

**Fig. 1:**
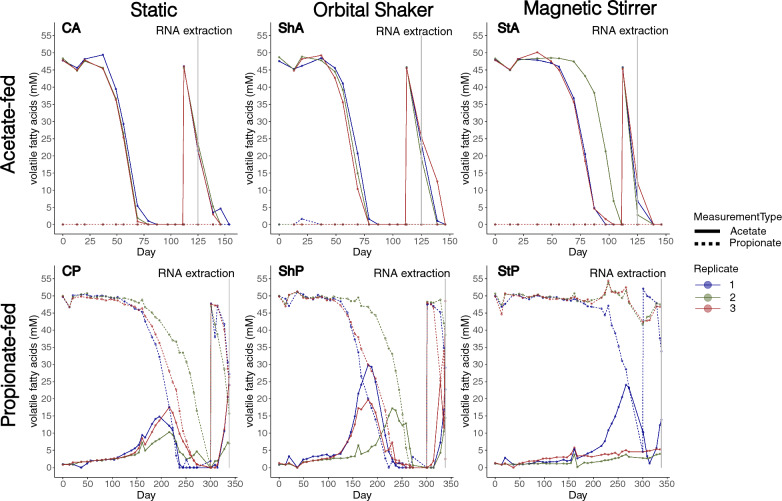
Degradation dynamics of acetate-fed (top) and propionate-fed (bottom) cultures subjected to different types of agitative motion: no motion (C, left), orbital shaking (Sh, middle), and magnetic stirring (St, right). Solid lines show the acetate concentration, while dashed lines show the propionate concentration (mM) and replicates are shown in different colours. For acetate-fed cultures, additional acetate (up to 50 mM) was added on day 111 and RNA extraction was performed on day 125. In the propionate-fed cultures, propionate was added on day 300 and RNA was extracted on day 338. Note the differing scales on the *x*-axes for acetate-fed and propionate-fed cultures

The acetate-fed cultures had similar degradation rates regardless of the type of motion but stirring extended the lag phase in StA cultures with 7–30 days as compared to cultures grown in static and shaken conditions (CP, ShP, Fig. 1, Table 1, Supplementary Data SE1 and SE2). In accordance with previous studies of the enrichment culture [19], the lag phase was considerably shorter for the second degradation (following the re-addition of 50 mM acetate, Table 1). This difference is likely due to cells and essential enzymes already being present at the point of re-addition. The slightly faster degradation observed in stirred cultures during the second degradation is likely explained by the shorter starvation period between the consumption of the initially supplemented acetate and its re-addition (Fig. 1).

**Table 1:** Cultivation metrices of acetate-fed (CA, ShA, StA) and propionate-fed (CP, ShP, StP) cultures subjected to different types of agitative motion: no motion (C), orbital shaking (Sh), and magnetic stirring (St)

Sample	First degradation					After substrate spiking	
	Substrate (50 mM)	Motion	Lag phase (days)	Acid deg. rate (mM/day)	Acetate accum. (mM)^a^	Lag phase (days)	Substrate conc. at RNA extraction (mM)
CA	Acetate	Static	49	1.70–1.77	–	0	21–24 (acetate)
ShA		Shaking	49	1.72–1.94	–	0	19–25 (acetate)
StA		Stirring	56–79	1.73–1.86	–	0	3–12 (acetate)
CP	Propionate	Static	181	0.40–0.70	19	0–13	16–24 (propionate)
ShP		Shaking	139–188	0.48–0.76	33	27–34	2–39 (propionate)
StP1^b^		Stirring	237	0.62	26	34	34 (propionate)

^a^Maximum acetate level during propionate degradation

^b^Only one of the triplicate propionate-fed cultures started to degrade propionate during stirring

In the propionate batch assays (CP, ShP, StP) the impact by motion on the lag phase was more pronounced. Strikingly, stirring had a negative impact on propionate degradation, as it only began in one (StP1) of the three replicates. The disruptive effect of stirring was further evident from the prolonged lag phase of over 50 days in StP1, compared cultures grown under static conditions (CP). Conversely, shaking motion (ShP) reduced the lag phase by approximately 40 days compared to the static condition in two of the replicates. Interestingly, once propionate degradation commenced, the degradation rates remained relatively similar across all conditions (Table 1). However, propionate degradation remained significantly slower than acetate degradation, consistent with previous studies on the enrichment culture when cultivated in a medium containing yeast extract (Supplementary Data SE2) [13, 19]. Furthermore, similar to SAO, the propionate degrading cultures all had shorter lag phases after substrate spiking compared to the first degradation (Table 1).

Contrary to the biphasic utilisation of propionate previously observed in enrichment cultures grown in yeast extract-supplemented medium [13], the present study revealed simultaneous degradation of acetate and propionate during the initial phase. This simultaneous degradation may be linked to the longer lag phase observed in the present study, likely due to the lack of crucial nutrients typically provided by the yeast extract. During this extended lag phase, the minimal degradation of propionate could allow the SAOB community to acclimate cultivation conditions, priming them for acetate degradation. As a result, once propionate degradation enters the logarithmic phase, the SAOB are already prepared to efficiently convert the acetate produced. Interestingly, despite the extended lag phase, the degradation rates were ultimately comparable between cultures with and without yeast extract supplementation once degradation had commenced (Supplementary Data SE2).

### CFD modelling and floc formation observation

**Fig. 2:**
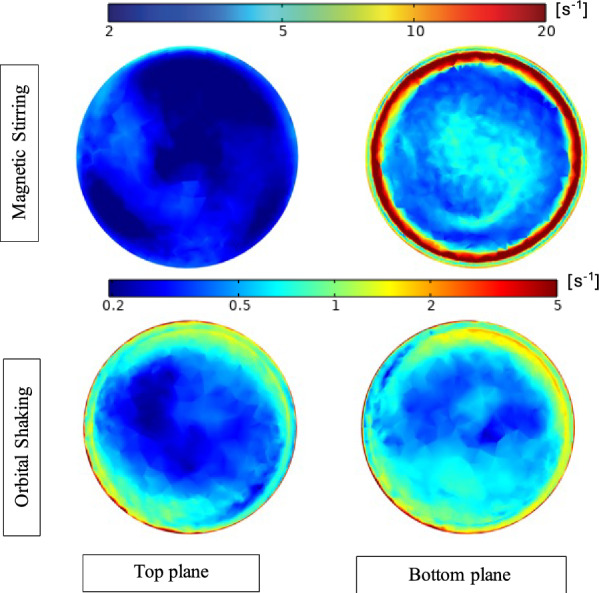
Contour plots depicting shear rates (s^−1^) for magnetic stirring (top plane: **a** bottom plane: **b** and orbital shaking (top plane: **c** bottom plane: **d**). Logarithmic scale has been used for both colour legends to highlight the variation in shear rates

The CFD modelling showed that the stirring motion generated high shear rates, which intensified with vertical depth as proximity to the magnetic stirrer increased, reaching a maximum of ~ 20 s^−1^ (Fig. 2). Contrastingly, the shaking motion resulted in relatively lower shear rates (maximum ~ 5 s^−1^) and showed minimal variation with vertical depth. Consequently, the hydrodynamic forces acting on solid particles were comparatively greater for magnetic stirring than for orbital shaking.

**Fig. 3:**
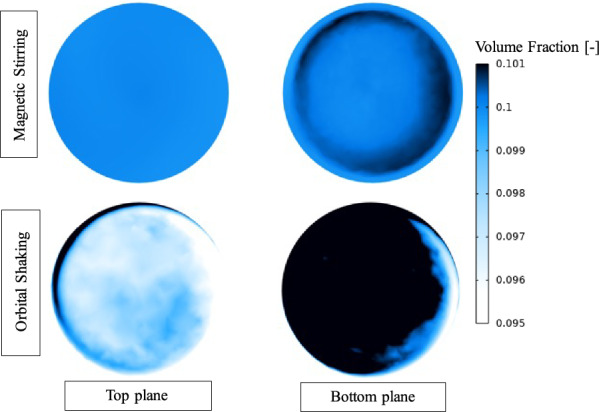
Contour plots corresponding to volume fraction of solid particles, unitless, with an initial value of solid particles 0.1 (1% of total volume) with homogenous distribution for both motions with magnetic stirring to the top and orbital shaking at the bottom. The deviation greater than 0.1 indicates regions with higher concentration of solid particles

Regarding the concentration of particles, mapping the volume fraction against the shear rate revealed that the highest solid particle concentrations occurred in areas with steep shear rate gradients, particularly near the bottom and along the walls (Fig. 3). During the shaking motion, a considerably higher volume fraction was observed at the bottom plane, likely due to the relatively weak but higher gradient shear rates that were generated by shaking, which were insufficient to counteract the negative buoyancy of the particles. The stirring motion gave rise to minor differences in particle distribution, mostly confined to the bottom of the liquid located close to the wall.

The results from the CFD modelling indicate that cells in the different setups were subjected to varying shear stress and fluid motion, which likely significantly impacted their ability to form and sustain cellular aggregates. For cell aggregation to occur, cells must be in close proximity to each other or to a surface, as this enables adhesive forces and the development of a shared extracellular matrix [21, 59]. In addition to motions influencing the initial cell aggregation, shear rates can also affect the size of the flocs. This is because higher shear rates impose an upper limit on floc size, leading to the formation of smaller flocs [60]. The present study demonstrated that stirring clearly prevented the formation of visible flocs due to its disruptive effect on aggregation.

Under shaking conditions, the enhanced volume fraction of particles (cells) likely increased the likelihood of initial cell–cell contact, while lower shear rates than in the stirring favoured the establishment of stable cell–cell adhesion. In line with these hypotheses, visual observations of the cultures revealed that shaking conditions resulted in the largest flocs (Supplementary note 1, Supplementary Fig. S1). This could be attributed to increased particle collision than under static conditions, facilitating the establishment of cell–cell adhesion. The observation aligns with previous studies which have demonstrated that moderate shear forces is necessary for the formation of a specific type of compact cellular aggregates, known as granules [61], and that turbulence promotes cellular aggregation and biofilm formation [62]. In addition, a study of a co-culture of syntrophic butyrate oxidising bacteria and a hydrogenotrophic partner showed that physical disruption through shaking or ultrasound treatment did not affect the tendency of aggregation [63].

For microscopic inspection, all samples were collected from the top phase of the culture assays to prevent any disturbance of the formed flocs and biofilms, favouring the sampling of platonic over aggregated cells. Nevertheless, aggregates were still observed in the shaking motion samples and, surprisingly, in one of the stirred replicates that was unable to degrade propionate (StP3, Supplementary Fig. S2). The presence of aggregates in the shaking motion samples, but not in the static control samples, aligns with the enhanced volume fraction of particles and the fluidic dynamics predicted by the CDF model under shaking motion. The presence of microscopic flocs in one of the stirred cultures implies that even under the high shear conditions, initial cell–cell adhesion and the formation of microscopic flocs consisting of a few cells is possible. However, although syntrophic relationships had been established by the second substrate addition in StA and StP1, the impact of shear stress under stirring conditions remained significant throughout the experimental period. Even after interactions were formed, microbial communities had to continuously withstand mechanical forces, potentially affecting their structural integrity, metabolic activity, and ability to establish new interspecies connections. Methanogenic autofluorescence was observed in all samples except StP2, which is one of the stirred replicates that was unable to degrade propionate.

Based on these results, it can be hypothesised that stirring and high shear stress impair propionate degradation more than acetate degradation due to the SPOB’s greater reliance on flocculation and proximity to the methanogenic partner compared to the SAOB. One potential explanation is that the SPOB may require closer proximity to the methanogenic partner for transferring reducing equivalents, such as through H_2_-mediated or direct electron transfer. If hydrogen serves as the primary carrier of reducing equivalents for the SPOB, the close spatial arrangement of the hydrogen producer and consumer within flocs or aggregates creates localised micro-environments, wherein hydrogen partial pressures are lower than the surrounding medium [64]. Stirring disrupts this arrangement, increasing the distance between consumer and producer, and thus elevates the hydrogen partial pressure that the producer is exposed to. It is possible that the SPOB is more sensitive to this elevated partial pressure than the SAOB. Another possibility is that SPOB are dependent on flocculation for protection to the high ammonia levels. A deeper understanding of how syntrophic organisms are affected by and protect themselves from high ammonia levels is needed to determine whether this is indeed the case.

For all stirred samples, it could be hypothesized that the higher shear stress enhanced fluid motion and reduced the boundary layer around microbial cells, improving mass transfer efficiency and allowing substrates to reach cells more rapidly. However, the negative impact of stirring on the acid degradation capacity of the communities, particularly the SPO community, suggests that any potential benefits from enhanced mass transfer were outweighed by the disruption of essential cell-to-cell interactions.

### Microbial compositions and dynamics

Approximately 87% of the total reads mapped to amplicon sequence variants taxonomically resolved to the species level. NMDS ordination plots on relative abundance showed a distinct separation between samples fed with acetate and those fed with propionate. However, no clear clustering was observed based on mode of motion (Supplementary Fig. S3 A). Instead, the degree of substrate degradation emerged as the primary determining factor (Supplementary Fig. S3B, C).

**Fig. 4:**
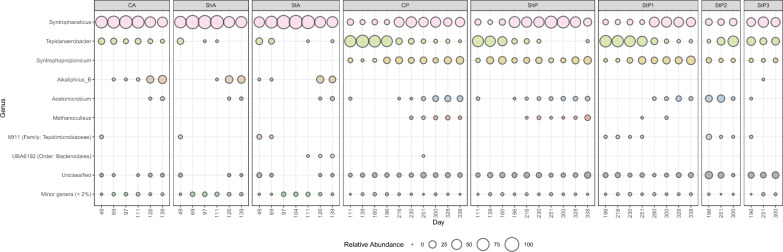
Bubble plot showing the relative abundance of microbial genera over time in acetate-fed (CA, ShA, StA) and propionate-fed (CP, ShP, StP1–3) cultures. All cultures, except stirred propionate-fed, show the relative abundance of merged counts across replicates. Stirred propionate-fed cultures (StP1–3) display individual replicate data instead. This distinction was made, because stirred propionate cultures exhibited substantial differences in ability to degrade propionate. For a detailed view of replicate-specific data for each condition, see Supplementary Figs. S4 and S5. Bubble size represents relative genus abundance, with genera with less than 2% relative abundance grouped as "Minor genera (< 2%)”

The microbial compositions and dynamics were similar across all acetate-fed cultures (Fig. 4, Supplementary Fig. S4). During the lag phase, known SAOB of the genera *Syntrophaceticus* (46–95%) and *Tepidanaerobacter* (13–29%) dominated. Once acetate oxidation had started, the relative abundance of *Syntrophaceticus* increased to > 90% for most of the replicates and remained relatively stable until the second addition of acetate. Members of this genus are frequently highlighted as the main SAOB in AD operating at high ammonia conditions [65, 66]. In the present study, an unclassified species belonging to the genera *Alkaliphilus* increased in relative abundance (from 0–3% to 11–49%) during this second acetate degradation, while acetate was still present in high levels. The activity of *Acetomicrobium* and *Alkaliphilus* in this study is particularly noteworthy, as these genera are frequently detected in biogas processes with high acetate levels, in association with syntrophic bacteria and hydrogenotrophic methanogens [67–69]. They have also been observed during hydrogen injection in power-to-gas applications [70], although their precise role in these systems remains unclear.

During propionate degradation in CP, ShP, and StP1, the microbial community was initially predominated by the genus *Tepidanaerobacter*, comprising over 50% relative abundance in most samples (Fig. 4, Supplementary Fig. S5). In addition, low to intermediate relative abundances of SAOB of the genera *Syntrophaceticus,* the SPOB candidate '*Ca*. Syntrophopropionicum' [12, 13, 19], *Acetomicrobium,* and an uncultured bacterial group belonging to the Family *Tepidimicrobiaceae* (referred to as Mt11) were observed. Once propionate oxidation was initiated, the relative abundance of '*Ca*. Syntrophopropionicum' increased to 46–77%. As acetate levels raised, so did the relative abundance of *Syntrophaceticus. Acetomicrobium* remained at low but persistent relative abundance throughout the degradation in the propionate-fed cultures, with an increasing trend toward the end of the experiment in some samples (CP1–3, ShP1, StP1). Following the second addition of propionate on day 300, the microbial community structures remained relatively stable, except for a few samples which showed a further increase in the relative abundance of '*Ca*. Syntrophopropionicum'*,* reaching higher abundances than those observed during the first degradation. The stirred samples that failed to degrade propionate (ShP2 and ShP3) exhibited microbial compositions similar to the lag-phase of the other cultures, with no observed increase in the relative abundance of the SPOB nor the SAOB as the experiment progressed*.*

qPCR analyses targeting the order *Methanomicrobiales* (including the genus *Methanoculleus*) revealed an abundance ranging from 5.52–8.50 log_10_ gene copies/ng DNA, while the total bacteria gene abundance was 8.68–12.27 log_10_ gene copies/ng DNA in the acetate- and propionate-fed cultures (Supplementary Fig. S6, Supplementary Data SE3). This indicates that the total bacterial community was consistently more abundant than the hydrogenotrophic methanogens (i.e., *Methanomicrobiales)* across all conditions. However, qPCR data showed relatively consistent presence of methanogens in all samples over the course of the study. Furthermore, while qPCR data showed similar abundance of *Methanomicrobiales* in both acetate- and propionate-fed cultures, this was not reflected in the 16S rRNA sequencing data (Fig. 4), where *Methanoculleus* was not detected in acetate-fed cultures.

### RNA extraction, sequencing, and differential expression analysis

Approximately 53% of raw metatranscriptomic reads were classified as rRNA and subsequently removed in-silico. Of the total mRNA reads, 71% mapped to the reference MAGs, with the majority mapping to the SAOB *S. schinkii* (34%), followed by the hydrogenotrophic methanogen '*Ca*. Methanoculleus ammoniitolerans' (32%), the SPOB '*Ca.* S. ammoniitolerans' (24%), and members of the genera *Alkaliphilus* (3%) or *Acetomicrobium* (2%) (Supplementary Fig. S7). PCA analysis of the transformed and normalised count data showed a distinct clustering based on substrate type for all samples. However, the PCA using only propionate samples showed a distinct clustering according to the mode of motion (Supplementary Fig. S8).

In the acetate fed samples, the SAOB *S. schinkii* exhibited the highest transcriptional activity, followed by the methanogen and *Alkaliphilus.* In propionate-fed samples, the methanogen and the SPOB displayed the highest activity, while *Alkaliphilus* was low in activity. The activity of SAOB was considerably lower in the propionate-fed samples compared to the acetate-fed, with moderate expression observed only in the stirred samples (StP) and one of the static control samples (Supplementary Fig. S7).

Based on the transcriptional activity and 16S rRNA gene-based microbial abundance, the MAGs for the syntrophic partners (SAOB, SPOB, methanogen) and *Acetomicrobium* were included in the differential expression analysis. As *Alkaliphilus* had low activity in the propionate-fed samples, this MAG was excluded in this analysis. All differential expression comparisons were made relative to the static control samples unless otherwise stated. For instance, the phrase “downregulated in stirred samples” indicates that the expression levels were lower in stirred samples compared to the static control. The term “motion” is used to collectively refer to the samples subjected to either stirring or orbital shaking. It is important to note that for stirred propionate-fed samples, RNA was extracted from two technical replicates of the same bottle (StP1), as the other two bottles lacked sufficient biomass for RNA extraction.

In the differential gene expression analysis, a total of 1396 differentially expressed genes (|log2fc|> 1.5 and p-adj < 0.05) were identified, mostly originating from the methanogenic MAG (Supplementary Fig. S9). For the other species, most differentially expressed genes were downregulated under both modes of motion. A Venn-diagram revealed a substantial overlap in differentially expressed genes between the two modes of motion for the methanogen and the SPOB, demonstrating that these changes occurred under both conditions (Supplementary Fig. S10). In contrast, the SAOB and the *Acetomicrobium* sp*.* showed less overlap, suggesting that the two motions induced regulation of a more distinct sets of genes in these species.

The following section discusses the differentially expressed genes related to core metabolic pathways, energy conservation, and microbial interactions and their surrounding genomic region. Emphasis has been placed on annotated genes, where surrounding genes in the genome exhibit a similar log-fold change. The data sheet containing the expression data and differential expression results, primarily used for analysis, can be found in Supplementary Data SE4.

### Main metabolic pathways and energy conservation

Consistent with previous studies of the enrichment culture [13], the transcriptomic data in the present study demonstrated that the SPOB '*Ca.* S. ammoniitolerans' expressed genes for all steps of propionate oxidation through the methylmalonyl-CoA pathway. The SAOB *S. schinkii* expressed genes for the Wood–Ljungdahl pathway, used in the reverse direction during syntrophic acetate oxidation [71], whereas the methanogen '*Ca*. Methanoculleus ammoniitolerans' expressed genes for hydrogenotrophic methanogenesis. These core metabolic genes were highly expressed by the species across all investigated conditions (Supplementary Data SE4).

**Fig. 5:**
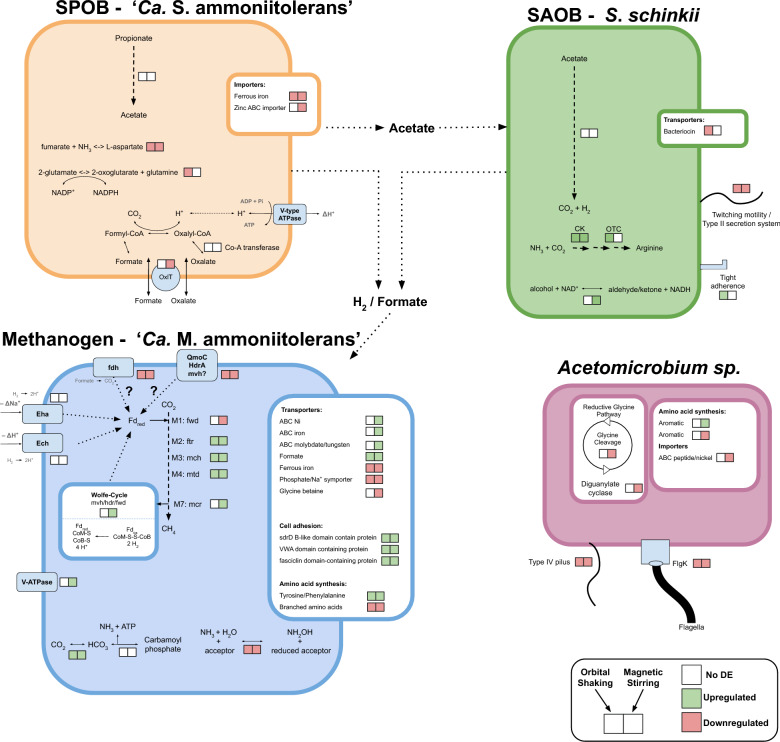
Graphical overview of the highlighted differentially expressed genes in stirred or orbital shaken samples relative to the static control samples. The red and green squares represent differentially downregulated and upregulated genes, respectively. White squares indicate that no differential change in expression was observed

For the SPOB, the shear forces induced by stirring caused a significant downregulation of an oxalate–formate antiporter (OxlT) located next to genes encoding a CoA-transferase (Fig. 5). In anaerobic bacteria, these enzymes have been shown to import oxalate (a divalent anion) by exchanging formate and, through a series of steps, convert the imported oxalate to formate, which is then exchanged for a new oxalate molecule, thereby closing the cycle. For each cycle, one intracellular proton is consumed, creating a proton gradient [72, 73] that could be used for ATP production (Fig. 5). This antiporter has previously been suggested to facilitate formate export in a syntrophic butyrate oxidising bacteria [74] or contribute to ATP production in syntrophic alkane-degrading bacteria [75]. In addition, prior research has linked increased expression of the oxalate–formate antiporter to faster propionate oxidation in thermophilic, ammonia-tolerant SPOB [14]. In combination with the higher expression of the oxalate–formate antiporter in aggregated states (static/shaking) in the present study, these findings suggest that this oxalate–formate cycling activity is dependent on cellular proximity and possibly requires interactions with other members of the syntrophic community. This activity could serve as a potential indicator of a well-performing SPOB community, and future culture studies of the SPOB may shed some light on this. Another question to address is the source of the oxalate in the culture medium, as no gene expression linked to oxalate production or transport was found in any of the other species in the culture.

For the SAOB, the gene expression of the reductive Wood–Ljungdahl pathway and energy conserving systems were relatively unaffected by the motions, which correlated with the observation of the relatively similar acetate degradation profiles of the batch assays. Rather, the most pronounced effects were observed for the methanogen, where both motions evoked upregulation of most of the genes involved in the hydrogenotrophic methanogenesis and a formate transporter. Furthermore, based on the genomic localisation of differentially expressed genes it appears that stirring induced the methanogen to increase the expression of genes involved in the so-called Wolfe cycle, a key energy conserving strategy in hydrogenotrophic methanogenesis (Fig. 5). The cycle plays a crucial role in regenerating reduced cofactors needed for hydrogenotrophic methanogenesis by coupling the exergonic reduction of heterodisulfide (CoM–S–S–CoB) to the endergonic reduction of ferredoxin, using H_2_. Alternatively, ferredoxin can be reduced through the energy-converting hydrogenases (Eha, Ech). During shaking, the energy-converting hydrogenase Eha was downregulated, with significant decreases observed in subunits *ehaB* and *ehaD.* Genes encoding the enzyme formylmethanofuran dehydrogenase (Fwd), which catalyse the first step of hydrogenotrophic methanogenesis, wherein reduced ferredoxin is consumed, were distributed at multiple different genomic regions (Supplementary Fig. S11). These fwd genes were located at distinct genomic loci, next to genes associated with either the Wolfe cycle, the energy-converting hydrogenases, or, intriguingly, next to a formate dehydrogenase. The formate dehydrogenase was downregulated under both motions, and the Fwd-gene downregulated in response to stirring. This co-localisation could suggest that the formate dehydrogenase is involved in ferredoxin reduction. However, formate has a redox potential similar to H_2_, and like hydrogen cannot reduce ferredoxin directly. To our knowledge, no such route of ferredoxin reduction via formate has been previously described. The Fwd encoding genes located next to the energy converting hydrogenase Eha were not differentially expressed. Furthermore, the methanogen upregulated a V/A-type ATPase during stirring, used to convert proton/sodium gradients to ATP, which can be related to the strive to maintain cellular activity during these harsh conditions.

In addition, both motions induced the methanogen to downregulate a quinone-modifying oxidoreductase, subunit QmoC. This enzyme is found in sulfate-reducing bacteria, where it is thought to play a role in the electron transfer from menaquinone to the terminal electron acceptor sulfate [76]. However, while QmoC is membrane-bound, our analysis indicated the differentially expressed QmoC to be cytoplasmic. The operon also contained a downregulated heterodisulfide reductase subunit (Hdr-A) and multiple hypothetical genes with similarities to iron–sulfur proteins, indicating involvement in electron transfer. A similar protein complex has been suggested to be involved in ferredoxin reduction through a QmoC/HdrA/Mvh-hydrogenase complex [77]. However, in the methanogen of this study, the operon lacked genes encoding the Mvh-hydrogenase, although genes for this enzyme were expressed and located elsewhere in the genome.

The transcriptomic activities of the *Acetomicrobium* sp. in propionate and acetate cultures and the *Alkaliphilus* sp. in acetate cultures were notable findings, considering the absence of any other sources for growth apart from the acids in the present study. These species expressed some genes of the Wood–Ljungdahl pathway but lacked key genes for the complete pathway. However, both species expressed genes related to the glycine synthase reductase pathway [78], suggesting its use for either acetate consumption or production. The *Alkaliphilus* MAG was not included in the differentially expressed gene analysis, as the expression by this MAG was negligible in propionate samples. A description on general expression and discussion on genomic potential for the *Alkaliphilus* MAG can be found in Supplementary note 2. Under stirred conditions, the *Acetomicrobium* sp. downregulated a glycine dehydrogenase and a dihydrolipoyl dehydrogenase, both components of the glycine cleavage system. They located within an operon that also contained a downregulated diguanylate cyclase, an enzyme regulating the ubiquitous second messenger cyclic di-GMP, which is an important regulator of bacterial behaviour, modulating processes such as biofilm formation and motility [79]. The reduced expression of diguanylate cyclase during stirring, when cells were more dispersed, suggests a possible link between glycine cleavage system activity and the flocculation state.

### Microbial interactions, motility, and biofilm formation

We expected that the motions, particularly stirring, would influence the expression for genes associated with motility, biofilm formation, and cell–cell interactions. However, in the SPOB no characterised genes linked to these functions were found to be differentially expressed. Nevertheless, the congregation of cells induced by orbital shaking caused the SAOB to upregulate an operon encoding tight adherence proteins (tadB, tadC) and pilus assembly proteins (CpaE, CpaF, CpaB). Expression of this operon was almost absent in static and stirred cultures. Some of these genes (tadB, tadC, CpaF) are known to be associated with the capability of colonisation of the mucus layer surrounding phytoplankton by oceanic bacteria [80], thus indicating a potential role in floc-formation of the SAOB during shaking. Shaking motion also caused the SAOB to downregulate an operon encoding ABC transporters involved in lipoprotein transport and the efflux of antimicrobial peptides. One of the genes in this operon showed BlastP similarity to bacteriocins, which are antimicrobial peptides that inhibit similar or closely related species, thereby offering a competitive advantage to the producer in the competition for resources and ecological niches [81]. Since cultures subjected to shaking motion formed the largest flocs, the downregulation of this bacteriocin system could represent an adaption to the close proximity to partner species on which they depend for metabolic activity.

Moreover, both motions caused the SAOB and the *Acetomicrobium* sp*.* to downregulate type IV pilus containing operons, including twitching motility proteins. Twitching motility allows bacteria to move over surfaces, and it is plausible that this mode of motility was only beneficial in the absence of shear stress and fluidic movement, as was the case for the static cultures. This is supported by the observation of surface-attached biofilms and biomass aggregation at the bottom of the culture bottles in static cultures, while both stirring and shaking seemed to prevent such formations. Furthermore, contact with surfaces has been shown to enhance the expression of type IV pilus in bacteria of the genus *Pseudomonas* [82, 83]. Therefore, it is possible that contact with the bottom of the bottle during static conditions contributed to the higher expression of type IV pilus in SAOB and *Acetomicrobium* sp*.* In addition to type IV pilus genes, the *Acetomicrobium* sp*.* downregulated a Flagellar hook-associated protein (FlgK) in response to both motions. The hook-associated protein plays a crucial role in connecting the flagellar hook to the flagellar filaments [84], suggesting increased motility under the static control conditions.

For the methanogen, both types of motions invoked upregulation of an operon containing numerous hypothetical proteins with BlastP similarity to “von Willebrand A (VWA) domain-containing protein”. While VWA domain-containing proteins are well-studied in eukaryotes, and are associated with cell adhesion and extracellular matrix proteins, less is known about their roles in prokaryotes [85]. The operon also included genes involved in methanogenesis (*mer* and *mtd*) of which *mtd* was upregulated. In addition, the operon contained genes encoding a Tubulin-like protein (CetZ), which in *Haloferax volcanii* regulates cell shape, contributed to the rod-shaped morphology needed for normal swimming motility [86]. However, considering that *Methanoculleus* species are irregular cocci this protein likely has a different role in this species. Furthermore, both motions evoked upregulation of unannotated genes, with BlastP similarities to proteins containing domains that indicate an involvement in adhesion and cell–surface interactions, namely: fasciclin-domain which is involved in ancient cell adhesion in plants and animals [87], PKD-domain found surface layer proteins of archaea [88], and SdrD B-like-domain which is important for cell adhesion [89].

### Transport, ammonia tolerance, amino acid, and other noteworthy gene expression changes

It is noteworthy that both the SPOB and the methanogen downregulated ferrous iron transport proteins under both types of motions. Ferrous iron is the predominant form of iron in anaerobic environments, and the downregulation of the transport systems may suggest that iron is a limited resource in the static control conditions, where mass transfer is determined by diffusion rates. In a separate experiment, the influence of iron level was tested under static conditions (data not shown). The addition of extra iron (ferric or ferrous 250 µM) seemed to have a negative effect on the syntrophic acid degradation, suggesting that iron deficiency is unlikely to be a major constraint in the current study. However, considering the interaction between iron and sulphide [90], it is possible that the iron addition inadvertently caused deficiency in bioavailable sulphur. Other differentially expressed transport proteins included downregulation of zinc transport under stirring by SPOB, whereas the methanogen upregulated ABC transport of nickel, iron, and molybdate/tungsten under stirred conditions and downregulated a phosphate/Na^+^ symporter under both motions. The *Acetomicrobium* sp. exhibited differential expression in genes for the transport of aromatic amino acids, with one operon downregulated during shaking motion and another upregulated under stirring motion. This suggests an adaption strategy to cope with the more challenging conditions associated with stirring. An ABC importer for peptide/nickel was also downregulated in stirred samples for the *Acetomicrobium* sp. (Fig. 5). In the methanogen, changes in amino acids metabolism included downregulation of genes involved in the synthesis of branched-chain amino acids (valine, isoleucine, leucine) under both motions. Conversely, several genes encoding phenylacetate-CoA ligase family protein, which are involved in phenylalanine and tyrosine metabolism, were upregulated under both motions.

Cultures were grown in high ammonia conditions (0.3 M) and many enzymes involved in ammonia-related reactions or osmotic stress showed differential expression. Notably, many of these enzymes exhibited similar expression patterns for both modes of motion. These systems could assimilate ammonia, thereby reducing intracellular ammonia levels, or to synthesise compatible solutes that help the cells manage ammonia stress. Furthermore, cellular aggregation could be employed by certain microbes to mitigate osmotic stress caused by the high ammonia [91]. If so, the disruption of aggregates through agitative motion, especially stirring, could undermine this strategy. This could explain the observed changes in expression between static and mixed cultures. For SPOB, both motions induced downregulation of aspartate ammonia lyase, converting ammonia and fumarate into L-aspartate. As fumarate is an intermediary of the methylmalonyl CoA pathway, the observed expression changes may reflect a shift in the SPOB’s metabolism toward catabolic processes in response to motion. This enzyme has also been shown to be upregulated in iron starved conditions in various bacterial species [92–95]. Moreover, the shaking motion evoked a downregulation of a NADPH dependent glutamate synthase in the SPOB, an enzyme that in conjunction with glutamine synthase is an important pathway of ammonia assimilation [96]. However, genes encoding glutamine synthase were located at other genomic locations and not differentially expressed. Glutamate is also an important counter-ion to potassium, a ion-pair which accumulates as a short-term response to osmotic shock [97]. However, given the long-term exposure to elevated ammonia levels, this function of glutamate is less probable for the species. Both motions caused the SAOB to upregulate a carbamate kinase, an enzyme that utilises ammonia and CO_2_ to produce carbamoyl phosphate. The operon also included an ornithine carbamoyltransferase, involved in arginine synthesis, which was upregulated in stirred cultures. In the methanogen, two operons with a potential role in ammonia assimilation were differentially expressed. The first operon included a carbonic anhydrase which converts carbon dioxide into carbonate, upregulated under both motions (Fig. 5). Surrounding genes encoded a carbamoyl-phosphate synthase, which combines bicarbonate and ammonia to form carbamoyl phosphate, an intermediary in pyrimidine synthesis and the urea cycle. The cluster also contained genes encoding a transport system for the compatible solute glycine betaine, which could indicate that the operon is involved in ammonia tolerance. The transport system was, however, downregulated in response to stirring, directly contrasting the carbonic anhydrase. The second operon was downregulated under both motions and contained a hydroxylamine reductase, which catalysed the reversible oxidation of ammonia to hydroxylamine (NH_2_OH), transferring electrons to an electron acceptor.

Finally, although certain differentially expressed genes were interesting, they did not fit into any of the previous categories. For the SAOB, the shear forces by stirring induced upregulation of two genes encoding a zinc dependent alcohol dehydrogenase, one of which was among the most highly expressed for the species. Alcohol dehydrogenases catalyse the reversible conversion of aldehydes/ketones to their corresponding alcohol while simultaneously oxidising NADH. In *S*. *schinki*, the enzyme has been proposed to partake in ethanol degradation in conjunction with acetaldehyde dehydrogenase, converting ethanol to acetyl-CoA, reducing NAD^−^ in the process [71]. This gene has been shown to be expressed by *Zhaonella formicivorans* in co-culture with a methylotrophic methanogen, utilizing the fourth mode of syntrophy, where methanol serves as an electron carrier [98]. However, this is unlikely to be the function of the alcohol dehydrogenase gene in *S. schinki*, as this species lacks several key genes for the methanol-producing pathway and was not associated with a methylotrophic methanogen in the enrichment culture. The high upregulation of this alcohol dehydrogenase containing operon is a notable finding, although its function and high expression remains enigmatic.

### Differences in gene expression between acetate- and propionate-fed cultures and potential coordinated cross-feeding of amino acids

The microbial composition varied between acetate- and propionate-fed cultures, with the primary distinction being the presence of the SPOB in the propionate-fed cultures and the *Alkaliphilus* sp. in the acetate-fed cultures. To investigate how both substrate type and microbial composition influenced gene expression in the SAOB, methanogen, and *Acetomicrobium* sp., a differential gene expression analysis was conducted (Supplementary Data SE5). This analysis compared the transcriptomes of all acetate-fed (three in total) with all propionate-fed cultures (nine in total). Changes in gene expression are reported relative to the propionate-fed cultures, with upregulation indicating higher expression in acetate-fed than in propionate-fed cultures.

Both the SAOB and methanogen downregulated several formate dehydrogenases and formate transporters in the acetate-fed cultures. Given that formate and hydrogen are considered as the primary carriers of reducing equivalents in syntrophic cultures, an upregulation of hydrogenases would typically be expected if the flux of reducing equivalents is to be maintained. However, no such upregulation was observed for the methanogen. Furthermore, the SAOB, contrary to expectations, downregulated hydrogenases [NiFe] 4e (ech) and [NiFe] 3B. These finding indicates that electron transfer in the acetate-fed cultures is more extensively routed through alternative pathways, such as direct electron transfer, or that the overall flux was reduced. For core metabolic pathways, gene expression remained largely unchanged, except for the upregulation of methenyltetrahydromethanopterin cyclohydrolase by the methanogen (step M3, Fig. 5).

Regarding pilus and flagellar systems, the SAOB downregulated most genes associated with the pilus system (pilABC), while genes involved in tight adherence and twitching motility were upregulated in the acetate culture. The methanogen downregulated the archaeal type IV pilus assembly protein (pilA), which is shown to be important for surface adherence [99]. These adaptions may contribute to the more pronounced surface attached biofilm growth observed in acetate-fed cultures (Supplementary Fig. S1).

Interspecies amino acid exchange is a particularly relevant aspect of the present study, as the metagenomic analyses indicated that all syntrophic microorganisms are auxotrophic (Supplementary Data, SE6 and SE7) and that they are cultivated under amino acid-limited conditions, aside from cysteine. The necessity of cross-feeding in the enrichment culture is further supported by the requirement of yeast extract by the SAOB *S. schinkii* when grown in pure-culture [30]. Experimental validation of auxotrophy in '*Ca.* M. ammoniitolerans' and '*Ca.* S. ammoniitolerans' awaits their isolation but SPOB such as *P. thermopropionicum* and several *Methanoculleus* species require the presence of yeast extract in mono-cultivation [100–102]. Still, our findings demonstrate that the propionate oxidiser is not essential for providing amino acids in acetate-degrading community, as these cultures managed to grow without yeast extract, even in the absence of the propionate oxidiser. Even though, no enrichment of amino-acid-related genes was observed for the methanogen, the SAOB had enrichment of upregulated genes involved in the synthesis of the branched-chain amino acids leucine and isoleucine in the acetate-fed cultures. The SPOB expressed the necessary genes for the leucine and isoleucine synthesis as observed in the propionate-fed cultures (Supplementary Fig. S12), indicating that the exchange of leucine/isoleucine from the SPOB to the SAOB is possible. However, these expression changes may not solely link to microbial interactions, as they could also result from the differences in acetate or propionate concentrations. For example, *Escherichia coli* can synthesise isoleucine by converting propionate to 2-ketobutyrate through an alternate pathway under anaerobic conditions in the presence of propionate [103].

In acetate-fed cultures, the SAOB exhibited enrichment of downregulated genes involved in threonine synthesis. Both the SPOB, the SAOB, and the *Alkaliphilus* sp. have the capability to produce threonine, complicating the ability to draw conclusions as to why the SAOB is downregulating its synthesis. Potential explanations include threonine provision by the *Alkaliphilus* sp. in acetate-fed cultures or overproduction and provision of threonine by the SAOB in propionate-fed conditions. Furthermore, the *Acetomicrobium* sp. showed enrichment of upregulated genes for tryptophan synthesis in acetate-fed conditions. This species had a complete set of genes for the synthesis of the tryptophan, unlike both the *Alkaliphilus* sp. and the SPOB, which lacked a complete set of genes. Taken together, the upregulation could indicate that *Acetomicrobium* sp. is providing the *Alkaliphilus* sp. with tryptophan in acetate-fed cultures. Tryptophan is a relatively costly amino acid to produce [104] and these amino acids have been shown to promote stronger auxotrophic interactions than the exchange of amino acids that are cheaper to produce [105]. Tryptophan has previously been suggested to play a role in coordinated cross-feeding between the SPOB *Pelotomaculum schinkii* and *Methanospirillum hungatei* [106]. However, given the complex and interconnected nature of interaction within these syntrophic communities further research is needed to confirm these findings.

## Conclusion

This study provides critical insights into how different mixing strategies affect syntrophic acid-degrading cultures, which play a key role in AD processes. Stirring substantially hindered initiation of syntrophic propionate oxidation while having minimal effect on syntrophic acetate oxidation. CFD modelling showed that stirring generated high shear rates and resulted in an even particle (cell) distribution, while shaking induced lower shear rates with more pronounced spatial gradients, causing cells to concentrate near the bottom. As a result, shaking reduced the lag phase and promoted the formation of larger microbial aggregates in the syntrophic propionate oxidizing community. This suggests that the movement, characterized by low shear stress, facilitated the initial connection and enhanced cell-to-cell interactions between SPOB and the methanogen.

Although motion had a limited impact on microbial community composition and the acid degradation rate, it triggered several key transcriptional changes in the syntrophic community (SAOB, SPOB, and methanogen), and the two species expressing the glycine synthase reductase pathway. Notably, stirring led to downregulation of an oxalate–formate antiporter in the SPOB. The higher expression of this system in the biofilm/aggregate forming cultures suggests a reliance on proximity for this activity, which may be linked to ATP production. Another notable effect was the upregulation of genes associated with hydrogenotrophic methanogenesis, particularly under stirred conditions, where the connection between the first and the last step in the hydrogenotrophic methanogenesis to regenerate ferredoxin appeared to have a prominent role. This has been suggested as an important strategy for methanogens for energy conservation. In addition, shaking caused the upregulation of tight adherence and pilus assembly genes in the SAOB, likely promoting cell aggregation. Conversely, both motions caused downregulation of motility-related genes in the SAOB and a *Acetomicrobium* sp., suggesting that motility was primarily beneficial under static conditions. Furthermore, pathway enrichment revealed potential cross-feeding of the branched amino acids leucine and isoleucine in propionate-fed cultures.

In particular, this study demonstrated that mixing and high shear stress disrupt the initial cell-to-cell connections between syntrophic propionate-oxidising bacteria and hydrogenotrophic methanogens, thereby negatively affecting the propionate degradation. These findings highlight the need to carefully control mixing strategies in anaerobic digesters, especially during periods of rising propionate levels when these communities need to establish themselves. Excessive shear stress may hinder the initiation of key syntrophic processes, particularly propionate oxidation, potentially leading to higher VFA accumulation and process instability. Conversely, moderate agitation that promotes microbial aggregation without excessive disruption could enhance syntrophic efficiency and biogas yield. These results have deepened the understanding of how hydrodynamic forces affect syntrophic interactions, which is crucial for optimizing anaerobic reactor design to maximize performance and stability, particularly under high-ammonia and high-VFA conditions. Furthermore, this knowledge could be leveraged in designing systems specifically aimed at producing propionate and other VFAs as end-products by adjusting mixing conditions to minimize syntrophic propionate degradation.

## Supplementary Information

Supplementary Material 1.

Supplementary Material 2.

Supplementary Material 3.

## Data Availability

All data generated or analysed during this study is included in this published article and its supplementary information files. The sequencing data generated and analysed in this study is available in the NCBI BioProject (PRJNA1187519). The 16S rRNA gene sequencing data have the SRA accession numbers SRR31396638-SRR31396766, and the metatranscriptomic sequencing data have the accession numbers SRR31439851-SRR31439861.
