# Supplementary material for: Disruption-induced changes in syntrophic propionate and acetate oxidation: flocculation, cell proximity, and microbial activity
Source: Biotechnol Biofuels Bioprod. 2025 Apr 19;18:45. doi: 10.1186/s13068-025-02644-3 (PMC12008871; doi:10.1186/s13068-025-02644-3)
Supplement: Supplementary file 1 — Supplementary Material 1. [file 13068_2025_2644_MOESM1_ESM.docx]

**Supplementary figures**

**Fig S1**


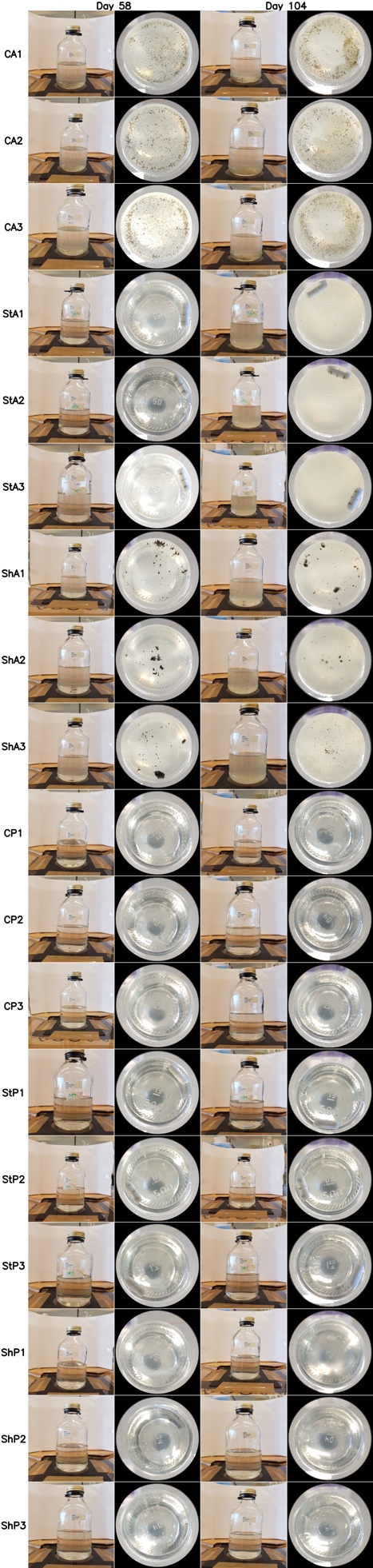


**Fig. S1** - Photographs of propionate- and acetate-degrading enrichment cultures exposed to different types of agitative motions. Images were taken on day 56 (left) and day 104 (right) of the experiment and were captured from the side and from below the serum bottles.

**Fig. S2**


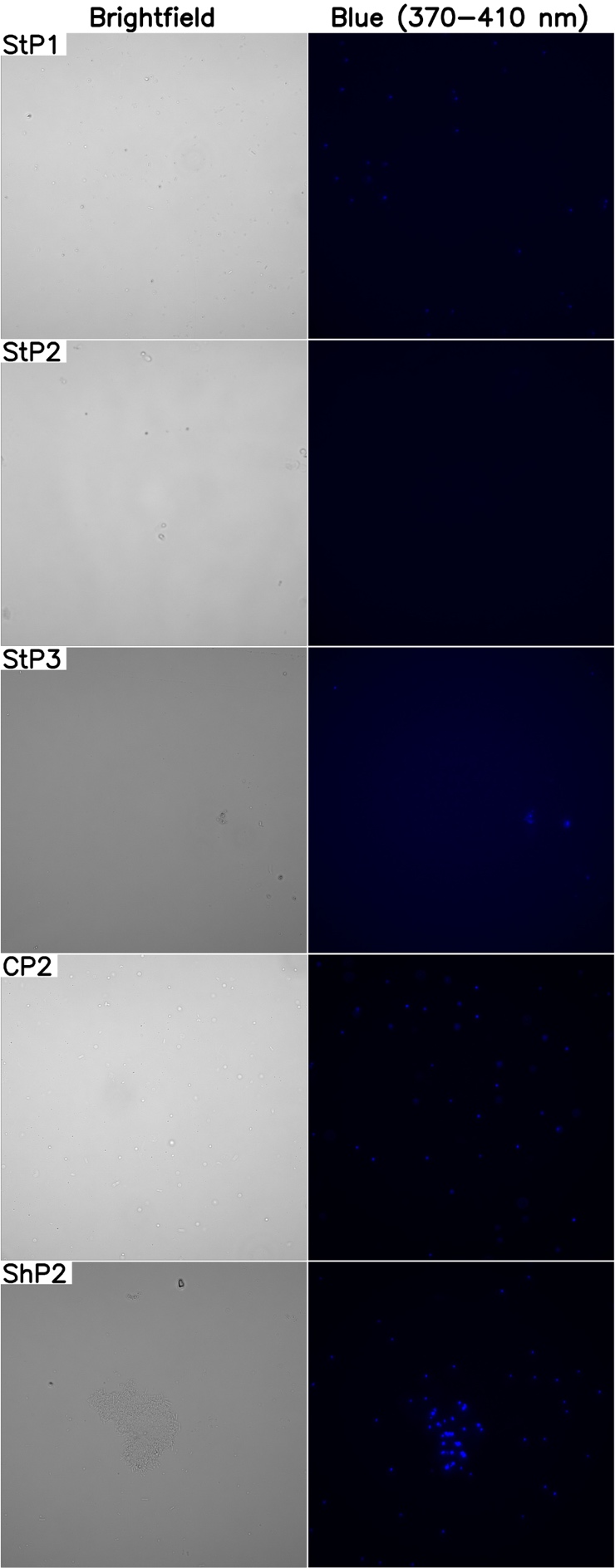


**Fig S2** – Micrographs of propionate-degrading cultures showing bright field microscopy (left) and methanogenic autofluorescence (left). Autofluorescence was captured with a 370–410 nm excitation filter and a 429–462 nm emission filter. All stirred samples were examined and visualized while only a representative sample were taken from the shaken and static cultures.

**Fig S3**

**Fig S3** - Non-metric multidimensional scaling (NMDS) ordination plot based on Bray Curtis dissimilarities of 16S rRNA gene abundance data. (A) NMDS plot of all samples with colours indicating substrate (red: acetate, blue: propionate) (B) NMDS plot of propionate-fed samples, and (C) NMDS plot of acetate-fed samples with colours indicating the day of the experiment and the shapes indicating the type of motion. Stress: A= 0.12, B= 0.09, C= 0.11

**Fig. S4**

**Fig. S4** - Relative abundance of microbial genera (bars) and concentration of acetate (black line) and propionate (blue) over time for acetate-fed samples. Each panel represent an individual replicate. CA (Static), ShA (Shaken), StA (Stirred)

**Fig. S5**

**Fig. S5** - Relative abundance of microbial genera (bars) and concentration of acetate (black line) and propionate (blue) over time for propionate-fed samples. Each panel represent an individual replicate. CP (Static), ShP (Shaken), StP (Stirred)

**Fig. S6**

**Fig. S6** **-** Quantification of 16S rRNA gene copy numbers using quantitative PCR with primers targeting Total bacteria (orange) and the order Methanomicrobiales (blue). The y-axis represents the log₁₀-transformed 16S rRNA gene copy number per nanogram of DNA. Error bars represent standard deviations from triplicate measurements, except for StA (Day 49), CA (Day 49), and CP (Days 139, 196), which are based on duplicates. StP includes data from a single replicate (StP1) only.

**Fig. S7**

**Fig. S7** – **Overview of metatranscriptomic counts for each sample and their distribution across MAGs. (A) Total raw counts (B) Percentage of counts mapped to each MAG, showing the relative contribution of each MAG. Detailed information about MAGs lacking descriptive names can be found elsewhere**^[[1]](#footnote-1)^**.**

**Fig. S8**

**Fig. S8** – Principal Component Analysis (PCA) on the regularised-logarithm transformed (rlog) metatranscriptomic data*.* (A) PCA comparing acetate- and propionate-fed cultures (B) PCA comparing different modes of motion (static, shaken, stirred) in propionate fed-cultures

**Fig. S9**

**Fig. S9** - Volcano plot depicting the differentially expressed genes between different motion conditions. Blue dots represent downregulated genes and orange dots upregulated genes. Rows corresponds to MAGs, and columns to the different modes of motion. Comparisons are made against static control samples as a baseline, except for the last column comparing orbital shaking against stirring. Differentially expressed genes are defined as |log2FC| > 1.5 and p-adj < 0.05.

**Fig. S10**


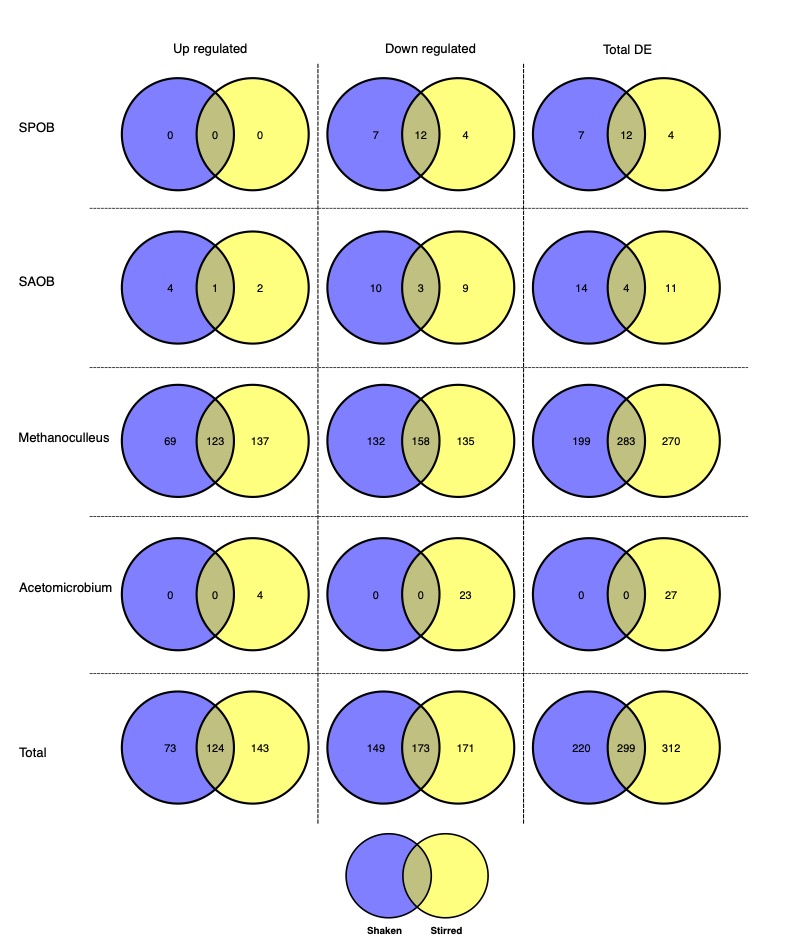


**Fig. S10** - Venn diagram showing the overlap of differentially expressed genes between orbital shaken (blue) and stirred (yellow) cultures compared to static control samples for the propionate-fed cultures.

**Fig. S11**

**Fig. S11** - Gene organisation of the clusters containing formylmethanofuran dehydrogenase subunits (fdr) for the methanogen *Ca*. Methanoculleus ammoniitolerans. Arrows indicate strand direction, arrow size the amino acid length of the coding sequence. Colours represents differential expression status: white (no change), red (downregulated), and green (upregulated), as compared to static cultures. Top and bottom arrow positions denote differential expression under stirred and shaken conditions, respectively. *ech*; ech hydrogenase, *eha*; energy-converting hydrogenase, *fdh;* Formate dehydrogenase, *ftr;* formylmethanofuran--tetrahydromethanopterin N-formyltransferase, *fwd/fmd*; formylmethanofuran dehydrogenase, *hdr;* heterodisulfide reductase, *hyp;* hydrogenase expression/formation protein, *mcr;* methyl-coenzyme M reductase, *moe;* molybdopterin molybdotransferase, *mvh/vhu/vhc*; F420-non-reducing hydrogenase, *rnc/DROSHA/RNT1;* ribonuclease III * Denotes sequential coding sequences with identical annotation, which have been merged for better visualisation.

**Fig. S12**

**Fig. S12** - Heatmap showing the expression of genes involved in amino acid synthesis, as identified by GapMind, for the propionate oxidiser *Ca*. S ammoniitolerans in propionate fed batch assays. Heatmap values show log2-transformed DESeq2 normalised counts. Genes with no expression are shown in grey. Row annotations include gene names and their corresponding amino acid abbreviations in parentheses.

1. Nils Weng et al., ‘Catabolism and Interactions of Syntrophic Propionate- and Acetate Oxidizing Microorganisms under Mesophilic, High-Ammonia Conditions’, *Frontiers in Microbiology* 15 (2024): 1389257, https://doi.org/10.3389/fmicb.2024.1389257. [↑](#footnote-ref-1)
