# Supplementary material for: Disruption-induced changes in syntrophic propionate and acetate oxidation: flocculation, cell proximity, and microbial activity
Source: Biotechnol Biofuels Bioprod. 2025 Apr 19;18:45. doi: 10.1186/s13068-025-02644-3 (PMC12008871; doi:10.1186/s13068-025-02644-3)
Supplement: Supplementary file 3 — Supplementary Material 3. [file 13068_2025_2644_MOESM3_ESM.docx]

**Supplementary note 1**

**Time-lapse imaging, visual and microscopic inspections**

Images were taken of the acetate and propionate degrading enrichment cultures (CA, CP, ShA, ShP, StA, StP) at day 56 and 104 (Supplementary Fig. S1). In the acetate-fed cultures (CA, ShA, StA), the static cultures formed biofilms attached to the bottom of the bottles and formed smaller microbial aggregates. Compared to the static cultures, the shaking motion (ShA) cultures formed larger microbial aggregates over the course of the experiment. In the stirred cultures, hardly any microbial aggregates could be observed. Unfortunately, images were not taken past day 104, which would have been needed capture the floc-formation of the propionate cultures during the exponential phase of propionate degradation. Nevertheless, visual inspection by eye indicated a similar trend of clear large spherical flocks formed under rocking motion, whereas static cultures formed dispersed biofilm along the glass surface of the bottom of the glass bottles. In contrast, stirred samples developed little to no visible microbial aggregates.

Fluorescent microscopic analysis revealed microbial aggregates showing methanogenic activities, as indicated by their auto-fluorescence (due to the presence of coenzyme F_420_) in the cultures used for transcriptomic analysis (Supplementary Fig. S2). At day 268, 0.1 ml samples were carefully collected from the upper level of the liquid medium to minimize disruption of aggregates or biofilms. The samples were then placed on microscope slides and covered with a coverslip for examination. This procedure likely favoured the sampling of platonic over flocculated cell. Fluorescence was detected in all cultures except in StP2 and small-scale microbial aggregates was observed in some of the cultures (StP3, ShP). Of the two non-propionate-degrading cultures StP2 and StP3, auto-fluorescence and small-scale aggregates were only revealed in StP3.

**Supplementary note 2**

***Alkaliphilus* sp. gene expression and its potential metabolic activity**

Transcriptional activity for MAG affiliated to the genus *Alkaliphilus* was only detected in acetate-fed cultures (CA, ShA, StA) but not in the propionate-fed cultures. As the differential expression analysis on the effect of agitative motion only was conducted on propionate-fed samples, this species was not included in that analysis. The following discussion presents a general overview of the genomic potential and expression profile of this species in the acetate-fed cultures, irrespective of motion conditions

*Alkaliphilus* *sp.* expressed all genes involved in the glycine reductase pathway (GSRP), in which formate is converted to acetate with glycine as a key intermediary [1]. In GSRP the glycine synthase system, glycine reductase, and the methyl branch of the Wood Ljungdahl pathway are utilized. In the *Alkaliphilus* *sp.* genome, key genes of the Wood Ljungdahl pathway which are not involved in GSRP, such as carbon monoxide dehydrogenase and the acetyl CoA synthase complex, were not encoded. This demonstrate that this species is not an acetogen that oxidise acetate by using the Wood Ljungdahl pathway in reverse direction when hydrogen and formate levels are kept at low levels by hydrogenotrophic methanogens. However, it has been proposed that GSRP could be operated in the in the oxidative direction to convert acetate to formate [1–4]. This would insinuate that the *Alkaliphilus* sp. compete with the SAOB for available acetate in the acetate-fed cultures. However, considering the lack of experimental evidence, this should be viewed as speculative. To get a better understanding of the directionality of the present species using GSRP, the genome was searched for acetate transporters and acetate permeases but no likely gene candidates were found. Consequently, the direction in which the GSRP is operated could not be determined in the present study.

The intermediary compound glycine could potentially also be converted to acetate via serine through the so-called reductive glycine pathway (RGP). However, only a few genes needed for this pathway were expressed, and genes serine hydro methyltransferase and serine deaminase were not found to be encoded in the genome. In addition to the GSRP, genes involved in the shikimate pathway for synthesis of branched amino acids were among the most highly expressed in all motion conditions. For motility the *Alkaliphilus* sp*.* expressed a few genes for type IV pili and none for flagella.

The fact that the *Alkaliphilus* sp. rapidly increased in relative abundance (Fig. 4 & S4) after the second spiking of acetate, and only in the acetate-fed cultures was an interesting observation. This contradict our initial theory that the microbe was a generalist, surviving on cell debris and increasing in relative abundance under times of acetate starvation [5]. One reason for this observation could be the higher pH at the end of the experiment [5], which would benefit alkaliphilic species, such as members of the genus *Alkaliphilus* [6,7]. Furthermore, in the present study the higher pH also increases the ammonia level, which could be favourable considering that ammonia is involved in the GSRP. This would be in accordance with previous study of the sulphate-reducing bacterium *Desulfovibrio desulfuricans*, which assimilates CO_2_ using GSRP. This study demonstrated an increased growth rate of *D. desulfuricans* with increasing ammonia levels (from 5.6 to 11.2 mM) and upregulation of genes related to ammonia limitation under autotrophic growth but not during heterotrophic conditions [1]. Consequently, in the present study the higher pH and/or higher ammonia level in combination with rapidly increased acetate level appear to be favourable aspect(s) for *Alkaliphilus* sp. expressing genes for GSRP. Other notable differences between the first and second degradation includes an overall higher cell density and more developed cellular aggregates and biofilms at the later stage. Moreover, the first degradation had higher presence of cysteine (or sulphuric compound derived from its degradation) and higher presence of trace elements as a result of the inoculation process, which could favour the SAOB *S. schinkii* over the *Alkaliphilus* sp. Further research is needed to confirm these hypotheses.

**References:**

1. Sánchez-Andrea I, Guedes IA, Hornung B, Boeren S, Lawson CE, Sousa DZ, et al. The reductive glycine pathway allows autotrophic growth of *Desulfovibrio desulfuricans*. Nat Commun. 2020;11:5090.

2. Zhu X, Campanaro S, Treu L, Seshadri R, Ivanova N, Kougias PG, et al. Metabolic dependencies govern microbial syntrophies during methanogenesis in an anaerobic digestion ecosystem. Microbiome. 2020;8:22.

3. Li C, Hao L, Lü F, Duan H, Zhang H, He P. Syntrophic Acetate-Oxidizing Microbial Consortia Enriched from Full-Scale Mesophilic Food Waste Anaerobic Digesters Showing High Biodiversity and Functional Redundancy. mSystems. 2022;7:e00339-22.

4. Kieft B, Finke N, McLaughlin RJ, Nallan AN, Krzywinski M, Crowe SA, et al. Genome-resolved correlation mapping links microbial community structure to metabolic interactions driving methane production from wastewater. Nat Commun. 2023;14:5380.

5. Weng N, Singh A, Ohlsson JA, Dolfing J, Westerholm M. Catabolism and interactions of syntrophic propionate- and acetate oxidizing microorganisms under mesophilic, high-ammonia conditions. Front Microbiol. 2024;15:1389257.

6. Postec A, Quéméneur M, Lecoeuvre A, Chabert N, Joseph M, Erauso G. *Alkaliphilus serpentinus* sp. nov. and *Alkaliphilus pronyensis* sp. nov., two novel anaerobic alkaliphilic species isolated from the serpentinite-hosted Prony Bay Hydrothermal Field (New Caledonia). Systematic and Applied Microbiology. 2021;44:126175.

7. Takai K, Moser DP, Onstott TC, Spoelstra N, Pfiffner SM, Dohnalkova A, et al. Alkaliphilus transvaalensis gen. nov., sp. nov., an extremely alkaliphilic bacterium isolated from a deep South African gold mine. Int J Syst Evol Microbiol. 2001;51:1245–56.
